# Gamma Transcranial Alternating Current Stimulation Enhances Working Memory Ability in Healthy People: An EEG Microstate Study

**DOI:** 10.3390/brainsci15040381

**Published:** 2025-04-07

**Authors:** Binbin Gao, Jinyan Zhang, Jianxu Zhang, Guangying Pei, Tiantian Liu, Li Wang, Shintaro Funahashi, Jinglong Wu, Zhilin Zhang, Jian Zhang

**Affiliations:** 1 School of Life Science, Beijing Institute of Technology, Beijing 100081, China; gao.binbin@bit.edu.cn; 2 School of Mechatronical Engineering, Beijing Institute of Technology, Beijing 100081, China; jinyanzhang@bit.edu.cn (J.Z.); jianxuzhang@bit.edu.cn (J.Z.); 3 School of Medical Technology, Beijing Institute of Technology, Beijing 100081, China; pei_guangying@bit.edu.cn (G.P.); tiantian2bit@bit.edu.cn (T.L.); 6120210008@bit.edu.cn (L.W.); wujl@bit.edu.cn (J.W.); 4 Advanced Research Institute for Multidisciplinary Science, Beijing Institute of Technology, Beijing 100081, China; funahashi@bit.edu.cn; 5 Research Center for Medical Artificial Intelligence, Shenzhen Institute of Advanced Technology, Chinese Academy of Sciences, Shenzhen 518055, China; 6 Department of Psychiatry, Graduate School of Medicine, Kyoto University, Kyoto 606-8501, Japan

**Keywords:** γ-tACS, working memory, microstate, resting-state EEG

## Abstract

Background:Working memory (WM) is a core cognitive function closely linked to various cognitive processes including language, decision making, and reasoning. Transcranial alternating current stimulation (tACS), a non-invasive brain stimulation technique, has been shown to modulate cognitive abilities and treat psychiatric disorders. Although gamma tACS (γ-tACS) has demonstrated positive effects on WM, its underlying neural mechanisms remain unclear. Methods: In this study, we employed electroencephalogram (EEG) microstate analysis to investigate the spatiotemporal dynamics of γ-tACS effects on WM performance. Healthy participants (N = 104) participated in two-back and three-back WM tasks before and after two types (sine and triangular) of γ-tACS, with sham stimulation as a control. Results: Our results revealed that γ-tACS improved performance in both the two-back and three-back tasks, with triangular γ-tACS showing greater accuracy improvement in the three-back task than the sham group. Furthermore, γ-tACS significantly modulated EEG microstate dynamics, specifically downregulating microstate Class C and upregulating microstate Classes D and B. These changes were positively correlated with reduced reaction times in the three-back task. Conclusions: Our findings establish microstate analysis as an effective approach for evaluating γ-tACS-induced changes in global brain activity and advance the understanding of how γ-tACS influences WM.

## 1. Introduction

Transcranial alternating current (tACS) is a non-invasive brain stimulation technique that applies specific frequencies of low-intensity currents to specific brain regions through electrodes which can achieve regulation of brain neural activity. As a form of transcranial electrical stimulation, tACS has the characteristics of high cost-effectiveness, easier application, and higher tolerance, demonstrating positive potential in treating various mental illnesses [1,2,3,4] and improving cognitive function [5,6,7].

Recent studies have focused on tACS applications for working memory (WM) [8,9,10]. WM is a limited-capacity memory system that can temporarily store and manipulate information required for complex cognitive tasks [11,12]. Clinically, WM impairments are observed in patients with mild cognitive impairment and psychiatric disorders such as schizophrenia, autism, and hyperactivity disorder [13,14,15,16]. The memory process is associated with brain oscillations such as delta, theta, alpha, beta, and gamma oscillations [17], and tACS theoretically provides oscillation frequencies related to neural discharges and affects the cognitive processes directly [18,19], but the underlying mechanism of tACS remains an open question.

Two meta-analyses have demonstrated that tACS can modestly to moderately improve cognitive function, with frequency-specific effects on different cognitive abilities [20,21]. α-tACS is more correlated with attentional control [22] and inhibitory control [23] compared with WM, and θ-tACS is linked with WM’s capacity, while the effects were opposite at different frequencies, with 4 Hz increasing the WM capacity and 7 Hz decreasing the WM capacity [24,25]. Meanwhile, β-tACS has been shown to improve short-term memory ability [26] and voluntary risk decision making [27], and γ-tACS enhances fluid intelligence [28] and significantly improves WM recall precision compared with alpha-frequency stimulation [29]. A behavioral study showed that γ-tACS regulation in the prefrontal area enhances the performance of participants in n-back tasks that require WM [30], indicating that γ-tACS has the potential to directly influence cognitive processes, but the explanation of its mechanism is not yet complete. The results of the analysis combined with electroencephalography (EEG) indicated that the superposition of γ-tACS significantly improves spatial WM performance [31]. Numerous studies have shown that γ-tACS has a positive impact on WM ability, and neurophysiological data help to better understand the potential mechanisms of γ-tACS effects.

Resting-state electroencephalography (EEG) is widely used to describe cognitive performance and evaluate cognitive function. The microstate analysis of resting-state EEG reflects the spatiotemporal dynamics of brain activity, reflecting the synchronous activity of neuronal populations within the brain [32,33]. By utilizing k-means clustering and establishing the ideal cluster count through cross-validation metrics, the preferred range for the number of maps across participants was found to be within 4–7 [34]. BOLD-fMRI analysis showed a significant correlation between resting-state microstates and brain functional networks [35,36,37]. Regarding the four typical types—Class A (right-frontal left-posterior), Class B (left-frontal right-posterior), Class C (midline frontal-occipital), and Class D (midline frontal)—a range of exciting results have been found [38,39,40,41]. The typical microstates have revealed the disorder of brain network organization and cognitive deficits in different stages of schizophrenia [42,43], and an elevated Class C in patients with schizophrenia showed a significant decrease after positive treatment outcomes [44]. The biomarker role of microstates is also reflected in healthy populations. In the mental arithmetic tasks, good performers showed a decrease in the Class C and an increase in Class D [45]. In WM research, the transition of microstates has been considered the core of WM function [46]. Therefore, it is reasonable for EEG microstates to play a significant role in the study of WM regulation mechanisms.

The aim of this study is to evaluate the regulatory effect of γ-tACS on regulating WM ability using EEG microstates. In previous studies, we characterized the changes in global brain activities before and after pure tACS using microstates [41]. On this basis, we applied microstates to the study of tACS regulation, targeting WM. Given that the activity of the prefrontal cortex is highly correlated with WM ability, we selected the bilateral dorsolateral prefrontal cortex (DLPFC) as the stimulation target. Participants underwent WM tasks before and after three types (sham, sine, and triangular wave) of γ-tACS, and their resting-state EEG signals were analyzed using the microstate method. We hypothesized that γ-tACS had an impact on the participants’ WM performance and microstate dynamics which was related to the type of stimulation. Furthermore, the association between the microstate and behavior parameters was investigated to find the patterns of brain activity dynamics under γ-tACS intervention.

## 2. Materials and Methods

### 2.1. Participants

This study involved 104 healthy participants between 18 and 37 years of age (mean age: 23.49 ± 3.14 standard deviation), including 64 males and 40 females. All participants reported normal or corrected-to-normal vision and were right-handed. The inclusion criterion was no history of neuropsychiatric diseases such as migraines, epilepsy, depression, or chronic insomnia. All participants received explanations of the experimental procedure and signed an informed consent form before the beginning of the study. After the experiment, all participants filled out a scale for their perception during the experiment period. This study followed the Declaration of Helsinki and obtained approval from the Medical and Experimental Animal Ethics Committee of the Beijing Institute of Technology (No. BIT-EC-H-2022080).

### 2.2. Experimental Procedures

**Figure 1:**
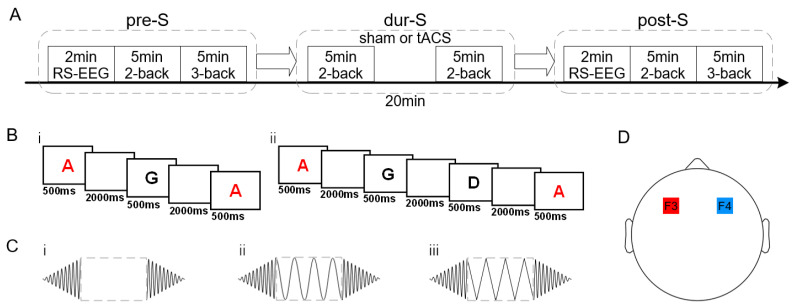
(**A**) Overview of the experiment’s design. (**B**) Overview of the behavioral experiments. (i) The two-back WM task. (ii) The three-back WM task. The letters appeared pseudo-random from A to J. (**C**) Three types of stimulus waveforms. (i) The sham stimulus. (ii) The sine wave. (iii) The triangular wave. (**D**) The stimulus electrodes’ setting positions. The two electrodes were applied at the bilateral DLPFC.

The experiment in this study adopted a randomized, single-blind, controlled experimental design. According to the different stimulus waveform conditions, all 104 participants were randomly divided into three groups, including 38 in the sham stimulation group, 28 in the sine stimulation group, and 38 in the triangular stimulation group. The experiment was conducted in an electromagnetic shielding room. Participants sat calmly and comfortably in the same chair. The experiment consisted of three main parts (Figure 1A). In the pre-stimulation (pre-S) period, 2 min of eyes-open resting-state EEG data were first collected, followed by a behavioral experiment consisting of a 5-min two-back task and a 5-min three-back task for the participants. During the stimulation (dur-S) period, participants received 20 min of electrical stimulation, during which the participants performed a 5-min two-back task twice (2.5–7.5 min and 12.5–17.5 min). The intra-stimulation WM task has shown an enhanced regulatory effect [30,47,48]. In the post-stimulation (post-S) period, the participants underwent the same experimental procedure as in the pre-S period.

The n-back task paradigm is one of the most popular paradigms in WM research [49]. The behavioral experiments in this study were controlled by an E-Prime 3.0 software (Psychology Software Tools, Pittsburgh, PA, USA) customized program. The experiment started with a cross at the center of the screen, which could remind participants to focus. A series of pseudo-random letters (A–J) was continuously presented at the screen’s center, and each letter appeared for 500 ms with an interval of 2000 ms (Figure 1B). Each block comprised 130 trials, of which 32 were targets. The participants were required to press the left mouse button when the letters that appeared were consistent with the second (two-back) or third (three-back) letter before them, and the maximum reaction time was specified to not exceed 2500 ms. The accuracy and response times (RTs) of the participants were used to evaluate the WM performance.

The developed electrical stimulator [50] was applied to provide three forms of stimuli (sham, sine, and triangular; Figure 1C) that met the research requirements. The stimulation duration was 20 min, the stimulation intensity was 2 mA from peak to peak, and the stimulation frequency was 40 Hz. The stimulator was set to fade in and fade out at the start and end of the stimulation for 30 s, respectively. After 30 s of ascending stimulation of the sham group, the stimulator was switched off to induce a typical tingling sensation [51]. The stimulation was transmitted through two electrodes (5 cm × 7 cm) which were placed at the bilateral DLPFC, corresponding to F3 and F4 of the international 10–20 system [52] (Figure 1D). Each electrode was covered with a sponge pad soaked in saline solution (0.9%) to reduce resistance and minimize discomfort during stimulation.

### 2.3. EEG Acquisition and Preprocessing

The Neuroscan system was used to collect 2-min resting-state EEG data during the pre-S and post-S stages, comprising 64 sintered Ag/AgCl electrodes, and it was arranged according to the international 10–20 system. The reference electrode was placed between Cz and CPz, and the ground electrode was placed between FPz and Fz. The impedances of the electrodes were maintained below 10 kΩ, and the data sampling frequency was 1000 Hz. The EEGLAB toolbox version 2021.1 (http://sccn.ucsd.edu/eeglab/, accessed on 19 September 2023) in MATLAB R2020b (Mathworks, Natick, MA, USA) was applied for data preprocessing. The average value of the left and right mastoid electrodes was used for re-reference, and the EEG data were bandpass filtered at 1–40 Hz. The EEGLAB toolbox provides a “runica” function for automated independent component analysis (ICA) decomposition [53], which can identify artifact components corresponding to eye movements, eye blinks, muscle artifacts, and cardiac artifacts. Ocular artifacts exhibit a strong frontal scalp distribution, being concentrated at a low frequency (below 5 Hz) power, as well as distinctive vertical and horizontal components. Muscle artifacts show a higher frequency (20 Hz and above) power and focal temporal topography. Cardiac artifacts contain a clear QRS complex at about 1 Hz, with a nearly linear gradient scalp topography and no peaks in the power spectrum. Components matching these criteria were removed through visual inspection prior to further analysis. The above preprocessing process conformed to the preprocessing steps proposed by the Organization for Human Brain Mapping [54]. The so-cleaned EEG data were further downsampled to 500 Hz.

### 2.4. Microstate Analysis

The MST1.0 EEGLAB toolbox was used to conduct the microstate analysis [55] while following a universal pipeline [32,56,57]. First, the global field power (GFP) was calculated, which represented the standard deviation of the voltages across all the scalp electrodes at a certain time and whose local maxima represented the instants of the highest field strength [58]. To ensure robustness, GFP peaks were selected with a minimum inter-peak interval of 10 ms, and outliers exceeding 2 standard deviations from the mean GFP amplitude were excluded. Next, the modified k-means algorithm was used to cluster these topographic maps, which disregarded voltage polarity to avoid redundant classification of inverse polarity patterns [33]. The microstate templates were calculated using four different cluster numbers (k = 4–7). For each k, the clustering process was repeated 50 times with random initializations and up to 1000 iterations to optimize convergence. The optimal k was determined based on the two criteria of the GEV and cross-validation criterion. Based on the computational results and previous studies [34,59], the number of microstate categories was determined to be four. Subsequently, the four microstate templates were back-fitted to the continuous EEG signals by calculating the spatial correlation between each template and the EEG topography at every time point. Each time frame was assigned to the microstate class with the highest correlation coefficient. To mitigate noise-induced fragmentation, transient microstate segments shorter than 30 ms were merged into adjacent states using global map dissimilarity criteria. Finally, four key parameters were computed for each microstate class.

For each participant, the four key parameters (duration, coverage, occurrence, and transition probability) of the four microstates were computed [60]. The duration, measured in milliseconds, represents the average length of time that a microstate remained stable. Coverage indicates the percentage of time records in which microstates dominated. Occurrence measures the average number of occurrences per second of a microstate. Finally, the transition probability refers to the percentage of transitions from one microstate to another over all transitions during a certain time.

### 2.5. Statistical Analysis

Demographic analysis was performed based on experimental grouping (sham stimulation group, sine stimulation group, and triangular stimulation group), univariate analysis of variance (ANOVA) based on age, and a chi-squared test based on gender. The behavioral performance data of the participants were analyzed using univariate ANOVA and a post hoc paired *t*-test. The WM behavioral performances of the participants were described in terms of accuracy and RT, and statistical analysis was conducted using paired *t*-tests and two-sample *t*-tests. Cohen’s d was used to evaluate the effect size [61].

After conducting microstate analysis, we obtained the topographies of four microstate classes (Class A, Class B, Class C, and Class D) for before and after the three stimulation types, with each template corresponding to four microstate parameters (duration, coverage, occurrence, and transition probability). To quantify the topographical similarity between the microstate topographies under different stimulation types, the +microstate toolbox was applied to conduct topographical ANOVA (TANOVA) [62]. For the microstate duration, coverage, and occurrence, we conducted two-way repeated measures ANOVA (rmANOVA) with the group and microstate class as factors [42]. When the main effects or interactions in the rmANOVA were significant, the post hoc pairwise Dunn’s test was employed. Data were expressed as the mean ± the standard error of the mean (SEM).

To compare the microstate dynamic differences between the pre-S and post-S stages in different stimulation groups, a paired *t*-test was conducted on the microstate parameters pre-S and post-S. Similarly, a paired *t*-test was employed to analyze the statistical significance of the 12 transition probabilities (A → B, B → A, A → C, C → A, A → D, D → A, B → C, C → B, B → D, D → B, C → D, and D → C).

To further investigate whether the microstate parameters were significantly correlated with the behavioral parameters, the linear model analysis was fitted between the behavioral accuracy and RT with EEG microstate parameters. This analysis was only used for microstate parameters with significant pre-S and post-S differences. To maintain consistency during the calculation process, the x axis (behavioral parameters) and y axis (microstate parameters) were obtained by calculating the rate of change.

The *p* values were corrected with the Bonferroni test (corrected *p* value < 0.05) to minimize the risk of type I errors. The statistical tests were conducted using R (version 4.3.1).

## 3. Results

### 3.1. Demographic Details

The 104 participants were randomly divided into three groups corresponding to three different stimuli, as 38 (aged 23.9 ± 3.3 years), 28 (aged 22.8 ± 2.4 years) and 38 (aged 23.5 ± 3.2 years) healthy participants were involved in the sham, sine, and triangular groups, respectively. No significant differences in age or gender across the groups were found (Table 1).

### 3.2. WM Performances

**Figure 2:**
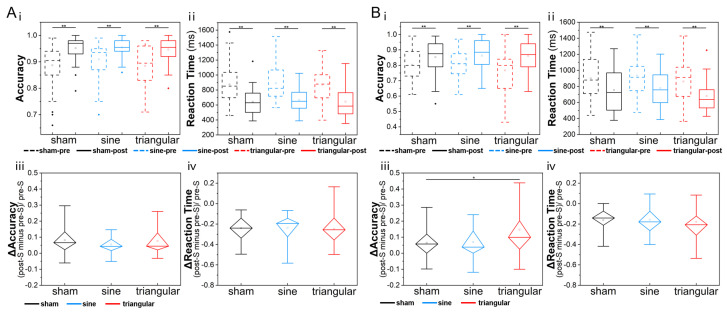
(**A**) Behavioral performances of participants in two-back WM tasks. (i) Accuracy comparison in pre-S and post-S stages for each group. (ii) RT comparison in pre-S and post-S stages for each group. (iii) The Δaccuracy across the groups. (iv) The ΔRT across the groups. (**B**) Behavioral performances of participants in three-back WM tasks. (i) Accuracy comparison in pre-S and post-S stages for each group. (ii) RT comparison in pre-S and post-S stages for each group. (iii) The Δaccuracy across the groups. (iv) The ΔRT across the groups. The black lines represent sham stimulation, the blue lines represent sine stimulation, the red lines represent triangular stimulation, dashed lines indicate the pre-S stage, and solid lines indicate the post-S stage (* *p* < 0.05; ** *p* < 0.01; Δ = (post-S − pre-S)/pre-S).

We analyzed the behavioral performance of the participants participating in WM tasks (Figure 2). Regardless of whether the participants were in the sham, sine, or triangular group, the accuracy was heightened and the RT shortened post stimulation, and all of the effects were statistically significant (all *p* < 0.001, paired *t*-test). The accuracy results were as follows: Cohen’s d (sham, two-back) = −0.52, Cohen’s d (sine, two-back) = −0.80, Cohen’s d (triangular, two-back) = −0.82, Cohen’s d (sham, two-back) = −0.46, Cohen’s d (sine, three-back) = −0.54, and Cohen’s d (triangular, three-back) = −0.78. The RT were as follows: Cohen’s d (sham, two-back) = 1.04, Cohen’s d (sine, two-back) = 1.13, Cohen’s d (triangular, two-back) = 1.14, Cohen’s d (sham, three-back) = 0.54, Cohen’s d (sine, three-back) = 0.65, and Cohen’s d (triangular, three-back) = 0.79. A two-sample *t*-test was applied to perform intergroup analysis on the changes in accuracy and RT. In the three-back task (Figure 2B), the change in accuracy for triangular group was significantly higher than that for the sham group (*p* = 0.039, Cohen’s d = −0.58, two-sample *t*-test; Figure 2B(iii)), while the change in RT showed no statistical difference across the groups (*p* > 0.05, Cohen’s d < 0.07, two-sample *t*-test). In the two-back task (Figure 2A), no statistical differences in the changes in accuracy or RT were observed in the intergroup comparisons (all *p* > 0.05, all Cohen’s d < 0.32, two-sample *t*-test).

### 3.3. Microstate Topographies

**Figure 3:**
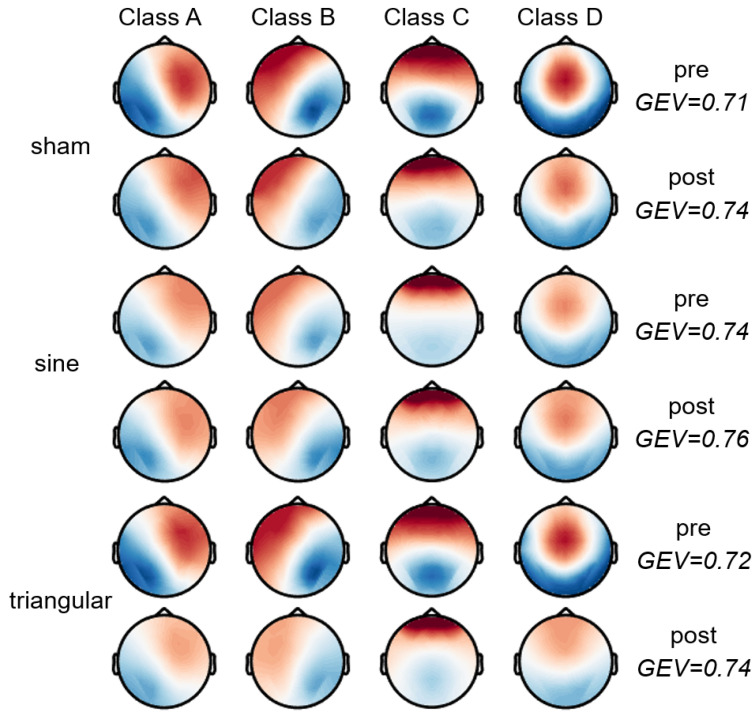
The four microstate templates under each stimulation condition. Color is used to depict the voltage, with red indicating positive values and blue indicating negative values. As the microstates were polarity-insensitive, the polarity could be inverted.

The archetypal four microstate patterns were calculated for each wave group in the pre-S and post-S stages (Figure 3), which were represented as Class A, Class B, Class C, and Class D based on other research studies [34,63,64]. Significant topographical differences between the templates under each condition were not detected through TANOVA. The GEV values were confirmed when the microstate template was back-fitted to the individual data of each condition (pre-sham: 0.71 ± 0.04; post-sham: 0.74 ± 0.05; pre-sine: 0.74 ± 0.06; post-sine: 0.76 ± 0.04; pre-triangular: 0.72 ± 0.04; post-triangular: 0.74 ± 0.05). When using two-sample *t*-tests, there were no significant differences found in the GEV values between the groups (all *p* > 0.05).

### 3.4. Microstate Parameters

Two-way rmANOVAs for the pre-S stage showed nonsignificant group × microstate class interaction in terms of duration (F(3,303) = 0.834, *p* = 0.544), coverage (F(3,303) = 0.206, *p* = 0.975), and occurrence (F(3,303) = 1.857, *p* = 0.088), while the analysis for the post-S stage showed significant group × microstate class interaction in terms of duration (F(3,303) = 4.093, *p* < 0.001), coverage (F(3,303) = 4.072, *p* < 0.001), and occurrence (F(3,303) = 11.04, *p* < 0.001). Post hoc pairwise group comparisons (Table 2) showed that the sine group had decreased coverage (*p* = 0.047) and occurrence levels (*p* = 0.049) for Class A compared with the sham group. The triangular group had increased coverage (*p* = 0.037) and occurrence levels (*p* = 0.002) for Class B, decreased duration (*p* < 0.001), coverage (*p* = 0.002), and occurrence levels (*p* < 0.001) for Class C, and increased duration (*p* = 0.010), coverage (*p* = 0.002), and occurrence levels (*p* < 0.001) for Class D compared with the sham group. For the sine and triangular groups, the duration (*p* = 0.034), coverage (*p* = 0.010), and occurrence levels (*p* = 0.005) of Class B, as well as the coverage (*p* = 0.019) of Class D, increased significantly. The duration (*p* < 0.001), coverage (*p* < 0.001), and occurrence levels (*p* = 0.001) of Class C decreased significantly.

**Figure 4:**
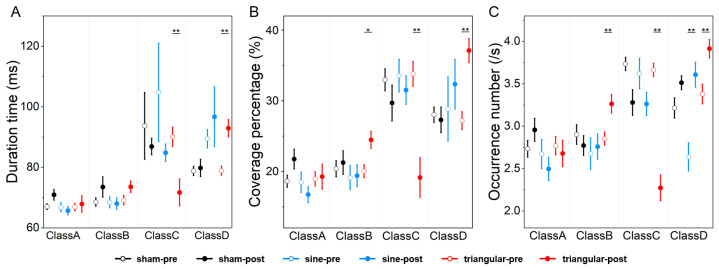
The statistical results for the (**A**) duration time, (**B**) coverage percentage, and (**C**) occurrence number under the three stimulation conditions. Error graphs show the mean ± SEM of the microstate parameters for the four microstates. The black, blue, and red lines indicate sham, sine, and triangular stimulation, respectively. Hollow circles represent the pre-S period, while solid circles represent the post-S period. The significance markers were the results of the paired *t*-test after Bonferroni correction (* *p* < 0.05; ** *p* < 0.01).

The comparison results for the microstate parameters (duration, coverage, and occurrence) pre- and post-stimulation under three different stimulation conditions (Figure 4) showed that the occurrence (*p* = 0.001) of Class D increased significantly post sine stimulation. For the triangular stimulation group, the coverage (*p* = 0.011) and occurrence (*p* = 0.007) of Class B increased. The duration (*p* = 0.001), coverage (*p* < 0.001), and occurrence (*p* < 0.001) of Class C decreased. The duration (*p* < 0.001), coverage (*p* < 0.001), and occurrence (*p* = 0.001) of Class D increased. No significant changes were observed in the microstate parameters between the pre-S and post-S sham groups.

**Figure 5:**
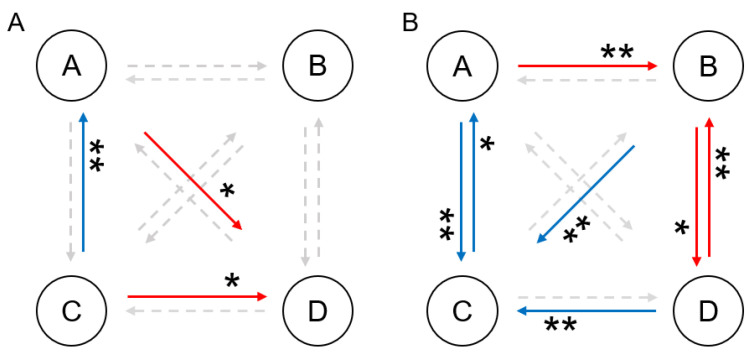
The statistical results of the transition probabilities for the four microstates of the (**A**) sine and (**B**) triangular groups. A red arrow indicates that the transition from one microstate to another was significantly increased compared with the pre-S stage, and a blue arrow indicates a significant decrease. The significance markers are the results of a paired *t*-test after Bonferroni correction (* *p* < 0.05; ** *p* < 0.01).

The statistical results of the transition probability changes in the four states of the different conditions are presented in Figure 5. There was no significant change in the transition probability between the sham pre-S and post-S periods. For the sine group, the transition probabilities of A → D (pre-S: 0.31 ± 0.21; post-S: 0.37 ± 0.18, *p* = 0.044) and C → D (pre-S: 0.39 ± 0.24; post-S: 0.45 ± 0.19, *p* = 0.027) significantly increased, while the transition probability of C → A (pre-S: 0.30 ± 0.13; post-S: 0.25 ± 0.09, *p* = 0.002) significantly decreased. For the triangular group, the transition probabilities of A → B (pre-S: 0.28 ± 0.08; post-S: 0.33 ± 0.09, *p* = 0.003), B → D (pre-S: 0.40 ± 0.11; post-S: 0.49 ± 0.14, *p* = 0.014), and D → B (pre-S: 0.30 ± 0.08; post-S: 0.41 ± 0.13, *p* < 0.001) significantly increased, while the transition probabilities of A → C (pre-S: 0.33 ± 0.09; post-S: 0.23 ± 0.15, *p* = 0.002), C → A (pre-S: 0.27 ± 0.08; post-S: 0.23 ± 0.10, *p* = 0.033), B → C (pre-S: 0.34 ± 0.09; post-S: 0.24 ± 0.16, *p* = 0.003), and D → C (pre-S: 0.40 ± 0.09; post-S: 0.28 ± 0.20, *p* = 0.002) significantly decreased.

### 3.5. Correlation Between Microstate and Behavior Parameters

**Figure 6:**
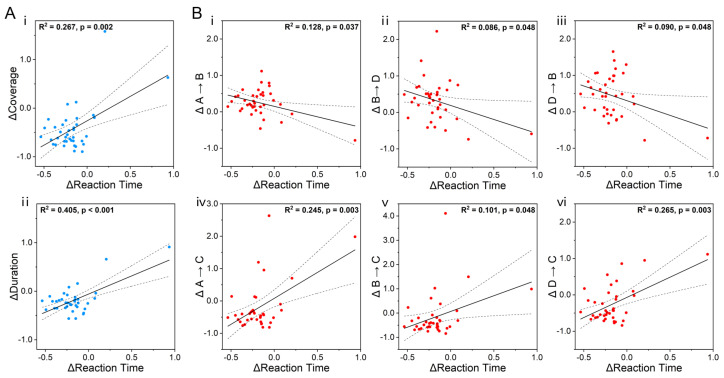
Correlations between triangular stimulation microstate parameters and three-back task behavior parameters. (**A**) The (i) coverage and (ii) duration of Class C were positively correlated with the RTs. (**B**) The transition probabilities of (i) A → B, (ii) B → D, and (iii) D → B were negatively correlated with the RTs, while those of (iv) A → C, (v) B → C, and (vi) D → C were positively correlated with the RTs. Each dot indicates the data of each participant. The range outlined by the dashed lines indicates the 95% confidence interval, and the solid lines represent linear regression. Δ = (post-S − pre-S)/pre-S.

Regression analysis was used to further calculate the correlation between the microstate parameters and behavioral parameters under different stimulus conditions. The duration (*p* = 0.002; R^2^ = 0.267) and coverage (*p* < 0.001; R^2^ = 0.405) of Class C were found to have a significant positive correlation with the RTs of the participants in the three-back task post triangular stimulation (Figure 6A). The transition probabilities were also found to have significant correlations with the RTs. Specifically, the transition probabilities of A → B (*p* = 0.037; R^2^ = 0.128), B → D (*p* = 0.048; R^2^ = 0.086), and D → B (*p* = 0.048; R^2^ = 0.090) had negative correlations with the RTs of the participants, and the transition probabilities of A → C (*p* = 0.003; R^2^ = 0.245), B → C (*p* = 0.048; R^2^ = 0.101), and D → C (*p* = 0.003; R^2^ = 0.265) had positive correlations (Figure 6B).

## 4. Discussion

The high enthusiasm for research on the impact of tACS on WM has sparked many discussions. Therefore, further work is needed to improve the understanding of how tACS affects WM. Brain-derived biomarkers hold promise in assisting us with evaluating the effect of γ-tACS on regulating WM ability. In this study, we conducted research using three types of brain stimulation (sham, sine, and triangular) on bilateral frontal lobe targets combined with recognized visual two-back and three-back WM behavioral paradigms. Our findings demonstrated that γ-tACS significantly improved WM performance. Compared with sham stimulation, both the sine and triangular stimulations notably influenced the EEG microstates. Furthermore, the post-S EEG microstate parameters following triangular stimulation exhibited significant changes which were correlated with behavioral outcomes. These findings provide valuable insights into the neural mechanisms underlying tACS-mediated modulation of WM.

In tACS studies on cognitive modulation, selection of the stimulation frequencies, stimulation targets, and stimulation waveform may lead to different outcomes. Studies have shown that gamma tACS is closely related to the maintenance and manipulation of information in WM, especially under high cognitive load, and is more suitable for regulating WM [30,65,66]. In terms of target selection, some studies focused on the parietal cortex or frontoparietal cortex to investigate visuospatial WM [24,25,67,68]. In our study, we chose the more widely used bilateral DLPFC [21], a region known for its central role in performing functions and WM maintenance. This choice could explain the significant improvements we observed in the two-back and three-back tasks [49]. In addition, compared with single-region modulation, bilateral modulation provides more robust regulation [8]. In terms of waveform selection, in addition to the sine waveform used in most studies, we incorporated triangular waveforms, which have clearer transitions and can more effectively entrain neural oscillations [69,70], allowing us to explore the impact of richer harmonic content on gamma oscillations.

The behavioral results indicate that γ-tACS enhanced the participants’ performances in WM tasks (Figure 2), confirming the positive effect of tACS, which is in agreement with previous studies [28,29,30,65]. In our study, the participants performed n-back tasks before and after stimulation, which might have led to improved performance in the sham stimulation group, but the effect of the sham group was lower than those of the sine and triangular groups. Moreover, for the higher-workload three-back task, in the inter-group comparison of behavioral changes, the accuracy improvement of the triangular group showed a significant increase compared with the sham group (*p* = 0.039, Cohen’s d = −0.58), indicating that the triangular wave group had the relatively best effect in regulating WM performance. Gamma oscillations are thought to reflect active processing of information, which may have facilitated maintaining the memory of the initially presented stimuli [71]. In other tACS studies, γ-tACS was shown to potentially enhance gamma power [72], thereby promoting improvement in the recall accuracy. Thus, γ-tACS can be effective in improving WM performance.

The resting-state EEG microstate analysis can extract information from unstructured EEG signals and is a powerful technique for studying the temporal dynamics of whole-brain neural networks, with high test-retest reliability [59]. Studies have shown that EEG microstates are closely related to the resting-state networks (RSNs) identified by functional magnetic resonance [35,36,37] and allow interpretation from the perspective of neuroscience. To our knowledge, we are the first to apply the biomarker effects of microstates to describe the mechanism by which tACS regulates WM. The modified k-means method was employed to extract the microstate characteristics in this study, and the most typical four microstate templates were defined (Figure 3). The GEV of the four microstate topographies under different groups was from 71% to 76%, which is a reasonable range [34]. Moreover, the TANOVA [62] showed no significant differences in the microstate topographies among the three conditions. Therefore, we obtained four typical topographical templates of Classes A, B, C, and D.

The four temporal parameters (duration, coverage, occurrence, and transition probability) of the four microstates can be used to quantify the dynamic changes in the brain [32]. Our research indicated that γ-tACS caused a significant increase in the coverage and occurrence of Class B, a significant decrease in the duration, coverage, and occurrence for Class C, and a significant increase in the duration, coverage, and occurrence for Class D. The microstates of Classes A and B are related to sensory processes, with Class A linking to the auditory resting-state network [35,37] and Class B correlating with the visual resting-state network and visual imagery thoughts [73]. Seitzman et al. reported that the variation in the Class B features depended on the presence or absence of visual input [74]. The microstates of Classes C and D are associated with the saliency network and dorsal attention network, respectively [35]. The results of Seitzman et al. showed that Class C was task-negative, and Class D was task-positive [74]. For participants who performed well in the mental arithmetic tasks, Class C decreased, and Class D increased [45]. In clinical terms, a recent study showed that the dynamics of Classes C and D are candidate endogenous phenotypes of schizophrenia [42], which is characterized by an increase in Class C and a decrease in Class D. The downregulation of Class C [44] and the upregulation of Class D [75] may be related to a positive impact on schizophrenia. Therefore, the impact of tACS on Classes B, C, and D may reflect the neural mechanisms by which it enhances WM performance.

In the present study, γ-tACS also triggered mutual transitions between microstates, where the transition probabilities (A → B, B → D, and D → B) were negatively correlated with the three-back RT, and the transition probabilities (A → C, B → C, and D → C) were positively correlated with the three-back RT. The microstate transition probabilities are nonrandom [60], which can reflect considerable cognitive changes [45]. Research on task-state microstates indicates that the drastic transition between Classes C and D is crucial for executing WM n-back tasks [46]. Complex cognitive tasks require cross-domain coordinated planning of the prefrontal control network [76], and the tACS of the prefrontal cortex might enhance this activity. Neuro-electrophysiological studies have found that WM tasks are related to neural oscillations of different frequencies. Specifically, the maintenance of WM information is related to γ oscillation [66]. As the workload of WM increases, the amplitude of the γ rhythms also increases [77]. Park et al. reported that high-gamma tACS can significantly increase WM capacity [78]. Triangular waves have more components in the spectrum and can generate higher frequency gamma stimulation, potentially explaining the more significant behavioral results and microstate parameter changes produced by triangular wave stimulation.

Although this study indicated that microstates can be used to investigate the internal neural regulatory mechanisms of tACS, several limitations should be noted. Our research focused on healthy young adults, subjectively excluding age and disease factors. Future studies could be conducted in a larger age range or by introducing clinical data to enrich research dimensions. EEG-fMRI simultaneous measurement will help to obtain more detailed information related to cognitive tasks, and the integration of machine learning algorithms can enhance the analysis of these multimodal data [43,79]. Moreover, our research concentrated on the immediate effects of a single γ-tACS session. Future studies could explore the potential of cross-frequency coupling stimulation [31,67,68,80], and the inclusion of a follow-up session [2,3] would allow for the evaluation of whether the cognitive benefits are sustained over time. This would provide valuable insights into the potential for long-term cognitive enhancement through tACS. Future studies could also expand the scope of cognitive assessment by including tasks that evaluate other domains, such as attention, executive function, or long-term memory. This would help determine whether the observed effects of γ-tACS are specific to WM or extend to broader cognitive functions. The current research has found that tACS downregulates Class C and upregulates Classes B and D, which may explain the potential mechanism of tACS in regulating WM performance.

## 5. Conclusions

We analyzed the WM performance and microstate parameters of healthy participants before and after participating in three types of γ-tACS. The results showed that γ-tACS improved WM performance, and the regulatory effect of triangular waves was better. The regulatory effect of γ-tACS was observed in the microstate Classes B, C, and D, with a significant decrease in microstate Class C and an increase in the microstates of Classes B and D. Consequently, microstate analysis serves as a crucial tool for assessing brain function related to tACS and is anticipated to elucidate its regulatory process.

## Figures and Tables

**Table 1:** Demographics of the participants.

	Sham	Sine	Triangular	$\chi^{2}/t$	*p*
	(n = 38)	(n = 28)	(n = 38)		
Age (years, mean ± SD)	23.9 ± 3.3	22.8 ± 2.4	23.5 ± 3.2	0.921	0.401 ^1^
Gender (F/M)	23/15	17/11	24/14	0.067	0.967 ^2^

^1^ Two-sample *t*-test. ^2^ Chi-squared test.

**Table 2:** Sham vs. sine vs. triangular groups for microstate parameters in post-S period and for each microstate class.

Parameter	Microstate	Sham	Sine	Triangular	Sham vs. Sine	Sham vs. Triangular	Sine vs. Triangular
		Mean ± SEM			*p*		
Duration (ms)	Class A	70.95 ± 1.75	65.72 ± 1.39	67.92 ± 2.74	0.829	1.000	1.000
	Class B	73.52 ± 3.36	68.04 ± 1.91	73.59 ± 1.91	0.431	0.738	0.034(↑)
	Class C	86.90 ± 2.72	84.84 ± 2.88	71.72 ± 4.43	1.000	<0.001(↓)	<0.001(↓)
	Class D	79.82 ± 2.86	96.70 ± 9.89	92.92 ± 2.87	0.402	<0.010(↑)	0.676
Coverage (%)	Class A	21.75 ± 1.40	16.73 ± 1.13	19.27 ± 1.76	0.047(↓)	0.352	0.997
	Class B	21.26 ± 1.66	19.40 ± 1.55	24.47 ± 1.21	1.000	0.037(↑)	0.010(↑)
	Class C	29.69 ± 2.53	31.52 ± 2.02	19.14 ± 2.80	0.572	0.002(↓)	<0.001(↓)
	Class D	27.30 ± 1.82	32.35 ± 3.50	37.11 ± 1.72	1.000	0.002(↑)	0.019(↑)
Occurrence (/s)	Class A	2.96 ± 0.14	2.50 ± 0.14	2.68 ± 0.15	0.049(↓)	0.453	0.842
	Class B	2.77 ± 0.12	2.76 ± 0.15	3.26 ± 0.11	1.000	0.002(↑)	0.005(↑)
	Class C	3.28 ± 0.15	3.26 ± 0.14	2.27 ± 0.15	1.000	<0.001(↓)	0.001(↓)
	Class D	3.51 ± 0.08	3.61 ± 0.15	3.91 ± 0.11	0.365	<0.001(↑)	0.123

The up or down arrows indicate significantly increased or decreased results for the corresponding parameters in the corresponding comparisons, respectively (corrected *p* < 0.05). The values of the microstate parameters (duration, coverage, and occurrence) are represented in the form of mean ± SEM.

## Data Availability

The data that support the findings of this study are available on request from the corresponding author. The data are not publicly available due to privacy or ethical restrictions.
